# Comment on: *European Heart Journal* global spotlight on European Medicines Agency evaluation of mavacamten

**DOI:** 10.1093/ehjopen/oeae013

**Published:** 2024-03-06

**Authors:** John A Spertus

**Affiliations:** University of Missouri—Kansas City’s Healthcare Institute for Innovations in Quality and Saint Luke’s Mid America Heart Institute, 4401 Wornall Road, 9th Floor, Kansas City, MO, USA

## Dear Editor,

I read, with interest, the summary of the European Medicines Agency’s (EMA) assessment of mavacamten for symptomatic obstructive hypertrophic cardiomyopathy (oHCM).^1^ In particular, I was concerned with the EMA’s interpretation of the benefits of mavacamten on the health status of patients: their symptoms, function, and quality of life. In the clinical trials reviewed by EMA, EXPLORER-HCM, and VALOR-HCM, patients’ health status benefits were quantified by the 23-item Kansas City Cardiomyopathy Questionnaire (KCCQ), a well-validated patient-reported outcome for various types of heart failure.

When analysing a continuous outcome, such as the KCCQ Clinical Summary Score (CSS), the mean differences in change over time between treatment groups are described and statistically compared. However, a recent review of how best to understand the clinical significance of differences in KCCQ scores between groups emphasizes that no patients necessarily experience the mean change observed across an entire population of patients.^2^ To better understand the *clinical significance* of health status differences between treatment groups, the proportion of individual patients who experience small, but important, moderate to large and very large changes in their health status should be described.^2^ With regards to the KCCQ, numerous studies, including in patients with oHCM, have documented that a 5-point change is small, but clinically important, a 10-point change reflects a moderate to large improvement or deterioration, and a change of more than 15–20 points is a very large change in patients’ health status.

The KCCQ CSS data from the EXPLORER-HCM trial showed marked differences in the proportion of patients treated with mavacamten who had either moderate or very large clinical improvements over 30 weeks compared with placebo-treated patients. The proportions with a ≥10-point improvement was 53.9% vs. 33.8% (absolute difference of 20.1%) and the proportion with a ≥20-point improvement was 36% vs. 13% (absolute difference of 23%), consistent with numbers needed to treat (100/Absolute rate difference × 100) of 4–5 for 1 patient to feel markedly better with mavacamten.^3^ This is a very favourable benefit profile and contrasts substantially with the comment by DeVries and colleagues that ‘it should be noted that the improvement in health status measured by the KCCQ-23 CSS (treatment difference 9.1 in EXPLORER-HCM and 9.5 in VALOR-HCM)…were just below the pre-defined clinically meaningful thresholds (increase of ≥10)…’. I believe that DeVries *et al.* have confused *mean differences between treatment groups* (9.1 and 9.5, respectively) with the thresholds of *clinically important change*  *for individual patients* (≥5, ≥ 10, and ≥20). Since our primary treatment goal in oHCM is to improve the health status of individual patients, a more proper conclusion should have been ‘a much larger proportion of patients had clinically important improvements in their symptoms, function, and quality of life when treated with mavacamten, as assessed by the KCCQ CSS’.
